# Can Entomopathogenic Nematodes and Their Symbiotic Bacteria Suppress Fruit Fly Pests? A Review

**DOI:** 10.3390/microorganisms11071682

**Published:** 2023-06-28

**Authors:** Jorge Toledo, Brenda M. Morán-Aceves, Jorge E. Ibarra, Pablo Liedo

**Affiliations:** 1 El Colegio de la Frontera Sur, Tapachula 30700, Chiapas, Mexico; pliedo@ecosur.mx; 2 Laboratorio de Reproducción de Hongos Entomopatógenos, Asociación de Cañeros del Soconusco, A.C. CNPR, Huixtla 30640, Chiapas, Mexico; breyma16ibt@gmail.com; 3 Departamento de Biotecnología y Bioquímica, Centro de Investigaciones y de Estudios Avanzados (CINVESTAV-IPN), Irapuato 36500, Guanajuato, Mexico; jorge.ibarra@cinvestav.mx

**Keywords:** fruit flies, entomopathogenic nematodes, biological control agent, sustainable approach

## Abstract

Fruit flies (Diptera: Tephritidae) are serious pests that affect fruit production and marketing. Both third instar larvae and pupae are biological stages that persist in the soil until adult emergence. Entomopathogenic nematodes (ENs) are biological control agents that are used to control agricultural pests in greenhouse or field conditions. Several studies have been carried out under laboratory and field conditions showing how ENs can be applied within an area-wide integrated pest management approach to control fruit fly species in orchards and backyard fruit trees. In this review, we analyze how soil physical characteristics and biotic factors affect the performance of these biological control agents. Of the reviewed papers, more than half evaluated the influence of soil texture, humidity, temperature, and other factors on the performance of infective juveniles (IJs). Abiotic factors that significantly influence the performance of IJs are temperature, humidity, and texture. Among the biotic factors that affect IJs are fungi, bacteria, mites, insects, and earthworms. We conclude that ENs have the potential to be applied in the drip area of fruit trees that are infested by fruit flies and contribute to their suppression. This approach, in conjunction with an area-wide pest management approach, may contribute to pest suppression and increase the sustainability of agroecosystems.

## 1. Introduction

The family Tephritidae (Diptera) includes species that constitute the most important fruit and vegetable pests worldwide. The family comprises ~4900 described species within 481 genera. Six of these genera (*Anastrepha*, *Bactrocera*, *Ceratitis*, *Dacus*, *Rhagoletis*, and *Zeugodacus*) contain ~70 species of agricultural importance [1,2,3,4,5]. These pests cause considerable economic losses because of direct damage to a wide range of crops [4,5]. In numerous countries, the development of fruticulture has been restricted by strict quarantines imposed by countries that import the produce and by the high costs associated with efforts directed towards the prevention, containment, suppression, and eradication of these pests.

To prevent or minimize the harmful effects of tephritid pests, growers must comply with health and safety standards required by the market, applying several cultural, chemical, legal, and biological control methods, including the sterile insect technique (SIT), which are applied using an area-wide approach [6,7]. Parasitoids can be released as part of the biological control of fruit flies, and this method can be enhanced by the application of entomopathogenic nematodes (ENs) and fungi [8,9,10,11,12,13,14,15,16]. It has been demonstrated that ENs have the potential to attack and infect the larvae of a wide range of fruit fly species and other pests and may thus represent an effective additional strategy when applied to the control of fruit flies in both conventional and organic agricultural production systems of tropical and subtropical fruits [14,17,18]. These ENs are produced at large scale and thus represent a cost-effective option for application as a suppression strategy mainly in domestic backyard fruit trees and marginal areas where the pest takes refuge outside of the crop season [10,14].

Entomopathogenic nematodes are organisms that naturally inhabit the soil and can persist in this habitat due to the wide range of hosts they can attack [19]. The principal route of infection by infective juveniles (IJs) is via natural apertures in the host, such as the spiracles [20], mouth, and anus [21], although some species can penetrate softer tissues such as the intersegmental joints. The juvenile stage, known as the “dauer” larva, is the infective phase that enters the host’s body and releases its symbiotic bacteria, causing a type of septicemia (putrefaction of fatty tissue) and consequent death of the host within two to three days following infection [22]. The juveniles feed on the putrefied tissue and continue their development until reaching the adult stage and reproduce within the host. The IJs of the family Heterorhabditidae are associated with bacteria of the genus *Photorhabdus*, and those of the family Steinernematidae with the genus *Xenorhabdus*, both of which belong to the family Gammaproteobacteria [22].

The use of *Steinernema* nematode species to pest suppression is already commonplace and has produced satisfactory results [17] against a number of pests. Their application has been reported in the suppression of populations of the root weevil (*Diaprepes abbreviatus* L.), a pest of citrus trees [23], the Japanese beetle, *Popillia japonica* Newman, and the sweet potato weevil, *Cylas formicarius* (F.) (Coleoptera: Apionidae) [24,25]. They can also effectively infect and kill larvae of the order Lepidoptera when applied in humid environments with moderate temperatures [26]. Similarly, it has been reported that various species of *Heterorhabditis* are effective and can be considered for the biological control of pest insects [27]. The infective capacity of nematodes on dipteran pests has been shown against larvae of *Musca domestica* (L.) [28], the cabbage root fly (Anthomyiidae, *Delia radicum* L.) [29], the onion fly (Anthomyiidae, *Delia antiqua* (Meigen), and the mushroom sciarid fly (Sciaridae, *Lycoriella auripila* Winnertz) [30].

In the case of fruit flies, nematodes mainly infect second- and third-instar larvae (early and late), as well as the adult phase during emergence, while infection in the pupal stage is low or null [9,31,32]. To obtain satisfactory results, it is necessary to optimize the application method of these biological control agents, considering the prevailing abiotic and biotic factors present in the regions where they will be applied, the susceptible stage of the pest, and the phenology of the crops that require protection. Moreover, it has been shown that their greatest potential may be mainly against pest populations of fruit trees in marginal areas, abandoned orchards (unmanaged), greenhouses, commercial orchards, and orchards under organic production. Recent reports have already documented the potential of ENs as biological control agents against fruit flies and other fruit pests [33,34]. Therefore, it is necessary to analyze the advantages they offer and their limitations for successful application under field conditions. The use of these biological control agents will contribute to improving pest management strategies and reducing the use of pesticides in the control of fruit flies in order to produce fruits that meet market quality and food safety requirements.

The main objective of this review was to document the potential offered by ENs of the families Heterorhabditidae and Steinernematidae by analyzing their interaction with biotic and abiotic factors, in terms of developing an effective application strategy that can function as an additional element in the area-wide management of fruit flies.

## 2. EN–Fruit Fly Interaction

### 2.1. Fruit Flies

Studies on the effect of ENs on fruit flies have been conducted on the third-instar larvae of different fruit fly species, such as *Anastrepha fraterculus*, *A. ludens*, *A. obliqua*, *A. serpentina*, *A. suspensa*, *Ceratitis capitata*, *C. rosa*, *Bactrocera dorsalis*, *B. litrofons*, *B. oleae*, *B. tryoni*, *B. zonata*, *Dacus ciliatus*, *D. curcubitae*, *D. dorsalis*, *Rhagoletis indifferens*, *R. cerasi*, *R. pomonella*, and *Zeugodacus cucurbitae* usually provided by laboratories that produce them using artificial larval diets according to the specifications established for each species and laboratory [9,35,36,37,38,39,40,41,42,43]. For some species, third instar larvae have been obtained from fruits infested by gravid wild females in controlled conditions [44,45] and others from infested fruits collected in field conditions for obtaining the larvae [10,46].

### 2.2. Nematode Rearing

Some nematodes evaluated in bioassays and applied directly in the field are reproduced through a rearing process, while others are obtained from commercial products distributed to control other pests. The rearing and multiplication of nematodes are conducted in vivo and in vitro following the criteria established in different methodologies. However, they have been conditioned to the requirements of each of the various studies conducted.

In vivo rearing has been conducted by inoculating greater wax moth, *Galleria mellonella* (L.) larvae, where infective juveniles (IJs) were collected using White traps [21]. The IJs produced by this process were used in laboratory bioassays, pilot tests, and for application in small agricultural cooperatives [47]. When a suitable host does not exist or when experimental conditions require it, in vitro rearing methods can be used. In vitro rearing methods allow for the development of nematodes without requiring a host, and they are also used for rearing aposymbiotic nematodes (without symbionts) [28].

**Figure 1:**
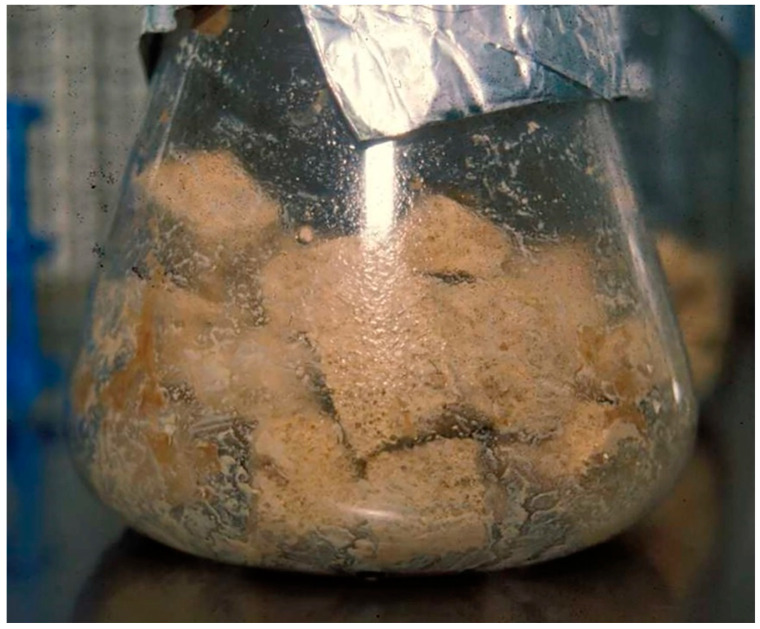
Production of *S. carpocapsae* using Bedding’s method. Foam cubes were imbedded in a paste made of cooked chicken livers, sterilized, and inoculated with *Xenorhabditis* sp. Five days later, the bacterial culture was inoculated with IJs. After several generations, IJs were migrated to the flask’s wall and collected.

In vitro production is conducted in both solid and liquid media. Monoxenic cultures are produced in solid media, where fermentation of the symbiont bacteria is first carried out, followed by the inoculation of IJs for their subsequent reproduction. When production using this process first began, Petri dishes were used with production media based on dog food (kibble), pig kidney, beef blood, and nutrient agars [48]. The Bedding method was subsequently developed [49], adopting the use of Erlenmeyer flasks with polyurethane foam, which were later replaced by sterilizable bags and a sterile air pumping system [47] (Figure 1).

Production in liquid media is conducted using monoxenic cultures. The concentration of symbiont bacteria prior to inoculation with IJs in the production medium is a crucial factor in the productivity of IJs, and in vitro production in liquid media is the most cost-effective process [50]. Although the industrial production of IJs of some species of nematodes is taking place, greater efficiency is still required through the standardization of processes and methods, since the processes are still trial and error.

In laboratory conditions, in vivo and in vitro techniques that are frequently used for rearing nematodes have been replicated as described and reported by several authors and are adapted to the requirements of each laboratory [28]. In in vivo breeding systems, all laboratories use White traps to harvest IJs that develop in live insects (generally *G. mellonella*) used as hosts. The harvested IJs are used in experimental evaluations and to maintain the nematode colonies [51,52]. Two types of media have been described in the in vitro rearing system, liver–kidney and lipids, which are used for the growth and successful reproduction of entomopathogenic nematodes with or without symbiotic bacteria. The liver–kidney agar system involves IJs to mature and reproduce without being associated with a host and is used to rear aposymbiotic nematodes [53]. However, a disadvantage of this rearing system is that it is easily contaminated due to the nutrients it includes, which promote the development of unwanted microorganisms. The lipid agar system is prepared by mixing the medium with nutrient broth, agar, and yeast extract in a 2 L Erlenmeyer flask, then adding distilled water, MgCl_2_•6H_2_O, corn oil, and corn syrup. Finally, ~20 mL volumes of sterile agar are placed in Petri dishes. The disadvantage of this method is that the inoculation of IJs and the harvest of nematodes are carried out under a laminar flow hood to reduce the risk of contamination of the medium, and the use of the nematodes produced depends exclusively on the requirements of the laboratory and/or project.

### 2.3. Commercial Production of ENs

The technology for the mass rearing of IJs has already been developed in several countries, including Mexico [54,55]. Based on standard requirements, namely, those conditioned by supply and demand, it is possible to reproduce IJs of all species at a mass scale [56]. Mass rearing is carried out in large bioreactors and managed to reduce costs and produce large quantities of ENs, ranging from 50,000 to 100,000 L in culture media. This approach is already used in Europe, North America, and Asia [57]. For the specific use of ENs in pest management programs, it should be noted that, although nematodes are easily produced both in vivo and in vitro in various complex semi-solid organic media, the cost of mass production using these methods represents a major constraint to their commercialization [57]. Therefore, development strategies and methodologies undergo slight adaptations according to the process adopted in each site. There are also factors that need to be improved, such as: (1) temperature, which is the easiest factor to control during the process; (2) oxygen concentration, temperature, and pH of the substrate used during the development of IJs; (3) humidity and aeration are more difficult factors to keep constant as the magnitude of mass rearing increases; and (4) the host density and inoculation rate should be estimated for each nematode species and host to optimize production [58]. Once these factors are controlled and standardized, labor costs can be reduced because the processes will be mechanized. In addition, with the mechanization of the process, a more stable and higher quality product should be obtained [59].

Efforts have also been intensified to develop new methods of mechanization of the process to improve in vivo rearing. One example is the “LOTEK” breeding system, which consists of perforated trays that allow for the automated collection of IJs for inoculation, incubating and harvesting IJs in 48 h, and a 97% carcass extraction efficiency [60]. The bases for the design of new separation processes have also been established, and the physical properties of the components have been described [61]. Despite the efforts that have been made, the technology still needs to be improved to optimize the separation process and scale up to a mass rearing system, which will contribute to substantially speed up the process, as in most cases it depends on sedimentation of the IJs by gravity. However, the technology that is being currently developed has been allowed to overcome the setback caused by the mass reproduction of IJs, and thus the priority now is to define a strategy with the greatest potential for field application, and thereby ensure the highest probability of success in terms of pest suppression. Additionally, an in vitro mass rearing method of IJs using solid monoxenic culture with four species of nematodes within the genera *Steinernema* and *Heterorhabditis* (*S. pakistanense*, *S. asiaticum*, *S. feltiae*, and *H. indica*), was described [62]. The method started by homogenizing and placing fresh chicken viscera on a porous foam (polyester polyurethane) substrate. Subsequently, they inoculated 10 mL of *Xenorhabdus* symbiotic bacteria culture in yeast extract (YS) broth. The production of IJs obtained for each species was: between 5 and 7 million per 500 mL flask for *S. pakistanense*, 4 and 5 million for *S. asiaticum*, 1 and 2 million for *S. feltiae*, and between 5 and 7 million for *H. indica*. The authors concluded that this large-scale production process may generate enough IJs with the quality required for applications under field conditions.

### 2.4. Evaluation of ENs on Fruit Flies

 **(a)**  **Bioassays**

The process begins by counting the number of IJs in a concentrated homogenized suspension, and then 1 mL is added to make a total volume of 100 mL with sterile water. The suspension is homogenized, and 1 mL is evenly distributed in one quadrant of a Petri dish. A stereoscopic microscope is used for quantification, and at least 10 counts (replicates) are performed per batch. The estimated number of IJs in a known volume is then used to make and adjust dilutions to the required densities for evaluation in the experiment.

The efficacy of these microbial control agents has been demonstrated by various laboratory bioassays and field trials. Several studies have been recently conducted against different fruit fly life stages (larvae, pupae, and adults), either exposed directly or indirectly via substrates [32,37,41,63,64,65,66]. These tests have been conducted in small experimental arenas, using sand, clay loam, or vermiculite as substrate. Other factors are also considered in these evaluations, including nematode origin, moisture content, temperature, and soil depth of hosts. Each experiment has been carried out independently from each other. The different developmental stages of the fruit fly species evaluated in bioassays are either from mass rearing or from infested fruits under forced laboratory conditions and from larvae that remain in infested mango, orange, and apple fruits [14,44,67]. The moisture content used in the different soil textures or substrates has been in the range of 10–30%.

 **(b)**  **Experiments simulating natural conditions**

Experiments that evaluate the efficacy of IJs under field conditions are relatively scarce; however, results from few studies that have been carried out indicate the potential of these organisms for the control of fruit fly larvae [14,68]. Therefore, the promotion of this strategy has merit, although it should be noted that evaluations have been conducted in experimental arenas of relatively small dimensions (100, 150, 400, 640, 710, and 2500 cm^2^) and in soils with controlled moisture content [10,13,14]. However, in a field study where IJs were applied directly on the soil in the drip area of trees using equipment adapted to a tractor, the results indicate the potential of IJs as biological control agents given the significant reduction in the number of adult flies emerging from these treated soils [46]. Similarly, Ref. [44] applications of IJs at a rate of 180 IJs/cm^2^ on infested fruits in experimental arenas with a surface area of 132 cm^2^ (12 × 11 cm), where each experimental unit contained 4 kg of sandy clay soil with a moisture content of 25% (*w*/*v* ratio) and three infested fruits were placed in the treated soil, have been tested [44]. Ten days after treatment, the soil was sieved to separate the larvae and to determine the infection level. Larvae that remained inside the fruits were collected with entomological forceps and observed to determine the infection. All the larvae and pupae were placed in Petri dishes containing moistened filter paper and dissected under a stereoscopic microscope to verify the presence of IJs and thus estimate the infection.

 **(c)**  **Effect of IJs combined with pesticides and other biological control agents**

In almost every case, the use of IJs combined with pesticides and other biological control agents has produced synergistic results when evaluated against fruit pests. When fruit flies were evaluated with IJs plus Spinosad, this resulted in a higher mortality of larvae of *C. capita* than with IJs or insecticide tested alone [69]. Moreover, when IJs have been applied with other insecticides, mortality in *C. capitata* pupae was higher or similar compared to IJs applied alone [66]. This synergistic effect provides greater efficacy to these control agents and prolongs the useful life of insecticides by delaying the development of resistance in the pests. Nematodes can also be applied in combination with entomopathogenic fungi such as *Beauveria bassiana* and *Metarhizium anisopliae*, since their efficacy is not reduced, and this method has shown to have a greater impact on the larvae, pupae, and adults of *Bactrocera zonata* and *B. darsolis* [70]. Additionally, the use of entomopathogenic fungi and nematodes separately was assessed against the larvae of *C. capitata* and showed that both biological control agents have a similar efficacy at the evaluated concentrations [71]. The combined applications of *B. bassiana* with *H. bacteriophora*, *H. megidis*, *S. feltiae* or *S. carpocapsae* against *C. capitata* resulted in synergistic interactions under laboratory conditions [72,73]. Additionally, when IJs of *S. feltiae* were applied alone and in combination with the conidia of three species of entomopathogenic fungi (*B. bassiana* (Bals.-Criv.) Vuill., *M. robertsii* JF Bisch, SA Rehner and Humber, and *Isaria fumosorosea* Wize) against larvae of *B. dorsalis*, higher mortality was recorded when they were applied together. Similarly, when two species of nematodes (*S. carpocapsae* and *S. riobrave*) were evaluated in combination with *B. bassiana*, *M. bruneum*, and *I. javanica*, a synergistic effect against *R. pomonella* larvae was observed [41]. However, there are other cases where there has been no indication of synergy, but there was also no effect of the fungi on the IJs [74].

 **(d)**  **Environmental conditions**

Entomopathogenic nematodes are natural inhabitants of the soil that live and persist in that environment, attacking many species of insect hosts that spend at least one stage of their life cycle in the soil. However, EN populations are affected by various biotic and abiotic factors, among which the main abiotic factors are extreme temperatures, low moisture content, and soil texture and pH. The main biotic factors are nematophagous fungi and acari, as well as some bacteria that can cause illnesses in nematodes. In the laboratory bioassays discussed in this review, Petri dishes and plastic containers of different sizes have been utilized as experimental arenas with filter paper, vermiculite, or soils of different textures as substrates. When filter paper is used, larvae or pupae used for tests do not serve as the diet or substrate. In contrast, when other substrate types, such as soils, are used, they must be sterilized beforehand in autoclaves to ensure the absence of any natural enemies of IJs. The textures of these soil types used as substrates are sandy, sandy loam, sandy clay, and clay. In general, the mean temperature used in these evaluations is 25 ± 1 °C. However, the range of temperatures reported is 15 to 35 °C. In studies conducted under field conditions, temperature is not controlled, but a range of 25 to 28 °C has generally been recorded [14]. In the case of environmental humidity, evaluations have been conducted in a range of 50 to 80% relative humidity (RH), with a mean value of 70 ± 5% R.H in most studies. However, interactions with abiotic factors, such as environmental temperature, soil texture and moisture content, and UV light, have also been analyzed. It is important to define the optimal soil moisture content, since it has been shown that high values of this parameter limit the displacement of IJs of *H. bacteriophora*. A soil moisture content of ≥30% has been observed to result in lower infection levels on *A. obliqua* larvae [75,76,77,78]. These results reflect the potential of these organisms against cryptic pests such as fruit flies, and a strategy that reflects the most effective application method must thus be defined, as well as the dose (density of IJs) required for application in the field.

### 2.5. Infective Capacity of Nematodes in Soil

The mobility of IJs is closely related to the texture and humidity of the soil. It has been observed that the infective capacity of *H. bacteriophora* on larvae of *A. obliqua* is higher in soils with a moisture content of 20, 25, and 30%. Despite slight variations in the levels of larval mortality, no significant difference was found among these moisture content values. In contrast, another study found that soils with a moisture content of 15% resulted in 40% mortality, while a moisture content of 10% resulted in mortality of 8%, which was the lowest value observed across all the different moisture content levels evaluated [74]. The same pattern was observed in *A. ludens* and *A. serpentina*, where the highest larval mortality by *H. bacteriophora* and *S. feltiae* was found in sandy clay soil with a moisture content of 15% and a temperature of 26 ± 1 °C [31,77,79,79]. Similarly, a mortality of 40% was observed in *A. obliqua* larvae in soil with a moisture content of 15%, while a moisture content of 25% resulted in 50% mortality [44]. Additionally, when IJs were applied directly on the soil with infested fruits placed on it and where IJs were applied directly on the infested fruits, this resulted in 12.3% and 16.6% larval mortality, respectively, and no significant differences were found between the two treatments [44].

There is evidence that when IJs are applied directly on infested fruits, there is an acceptable level of infection in both the larvae that are still inside the fruits and those that have left the fruits to pupate on the ground. However, the highest mortality by EN infection has been observed when IJs were applied on the soil at a density of 180 IJs/cm^2^, where a mortality of 27.5% was recorded in larvae recovered from the soil and extracted from the fruits. Infected juveniles applied directly on the fruits resulted in a mortality of 24.6%, both in larvae that had emerged to pupate in the soil and those extracted from the fruits. Similarly, IJ performance differs between different soil textures, as has been observed using *A. ludens* and *A. obliqua* fruit flies, where the best performance was recorded in sandy clay soil [31,32,79,80]. Every day, new species with potential as biological control agents against larvae and pupae of *C. capitata* are reported, such as *H. noenieputensis* and *S. yirgalemense* [81].

The effect of these two application methods was evaluated under field conditions on 36 fruits infested by *A. obliqua*, where a total of 423 pupae were obtained from soil treated with IJs that were applied on infested fruit, and 52 infected larvae were obtained from the fruits, which determined a 12.3% infection in larvae. However, when IJs were sprayed directly on the infested fruits, a total of 570 pupae were obtained from the soil, and 95 larvae were extracted from the fruits, resulting in a 16.7% infection in larvae obtained from fruits [44]. A similar strategy was proposed to control *B. dorsalis* and other species of fruit flies with ENs in sub-Saharan Africa [21].

## 3. Factors Affecting Infectivity

 **(a)**  **Abiotic factors**

During the development of the fruit flies’ life cycle, larvae and pupae are biological phases that occur in soils with different textures and moisture content and at different depths [82,83,84], which are optimal environments for the survival of EN IJs and for effectively attacking fruit fly larvae. For IJs to provide greater control of these pests, it is necessary to determine the influence of the factors that may limit their infective capacity. It is also crucial to consider the physical aspects that may limit the performance of IJs in the sites where they will be applied, among which the main ones are those associated with soil, such as humidity, texture, temperature, pH, and solar radiation. Therefore, it is necessary to determine the interaction between ENs and abiotic factors [14,32,40,85,86]. The first report of naturally occurring attacks by the nematode *Neoaplectana* (*Steinernema*) sp. was against *R. pomonella* larvae in Massachusetts, USA [87]. Subsequently, the nematodes were experimentally tested [41,42].

Soil texture is closely associated with soil moisture content, and this factor has been found to directly impact the movement of IJs of *H. indica* [31,32,88]. Thus, the performance of IJs is directly dependent on soil texture, as observed in IJs infecting the larvae of *A. obliqua*, which performed better in soil with sandy clay texture compared to sandy and sandy loam soils, all with a moisture content of 10% [75]. However, the foraging strategy of the nematodes must also be considered. For example, the “dauer” larvae of some nematode species, such as those of the genus *Heterorhabditis*, detect their host while moving randomly through the soil, while other species, such as *S. carpocapsae* and *S. feltiae*, and other *Steinernema* spp., ambush their host in the upper soil layers, and other species use both strategies (*Heterorhabditis* spp.). In addition, higher larvae infestation levels have been recorded in soils with a moisture content of 20, 25, and 30%, but the infective capacity of IJs has been observed to decrease with lower soil humidity. It has been reported that the IJs of *H. bacteriophora* have a greater capacity to move towards their host in sandy soils when the substrate has a higher moisture content [31,80]. Soil texture, moisture content, depth, and compression all influence the mobility of IJ hosts, as is the case of the larvae of several fruit fly species, where the soil depth where pupation occurs has been found to be related to these factors [82,89]. Soil texture may thus favor or limit the infective capacity of IJs [90].

Soil texture also influences the infection level caused by IJs to a great extent. Evaluations of different soil textures under laboratory conditions showed that soils with a sandy clay texture exhibit the greatest displacement of IJs of *H. bacteriophora* and *S. feltiae*, and the IJs in these soils also cause higher larval mortality compared to other soil textures [31,79]. Some EN species show a behavior similar to that observed in *H. indica* [91], and an evaluation of the vertical displacement and infective capacity of *Neoplectana* (*Steinernema*) *carpocapsae* in substrates with four different textures (pure silica sand, sandy clay, sandy loam, and clay) showed mortalities of 62.9, 58.6, 41.9, and 13.2%, respectively. These results indicate that the infective capacity of IJs when attacking and infecting the pupae of *G. mellonella* appears to decrease as the clay content of the substrate increases.

Entomopathogenic nematodes have a wide host range but, due to their excellent foraging ability, they have a low impact on non-target organisms. They also have a high reproductive rate, and can thus be mass-produced at a low cost. Their application in the field is compatible with various control methods already used for other agricultural pests [92]. 

Under laboratory conditions, satisfactory results have been reported in evaluations with larvae and recently emerged adults of different fruit fly species, such as *C. capitata*, *B. oleae*, *B. dorsalis*, *A. ludens*, *A. fraterculus*, *A. serpentina*, and *A. obliqua* [14,88,93,94,95,96,97,98]. Moreover, the species *S. riobrave* has been shown to cause high mortality in the third-stage larvae of *A. ludens* [99]. Additionally, bioassay-based characterizations of other ENs species are being carried out to determine their potential as biological control agents; as in the case of *Oscheius tipulae* tested against *C. capitata*, ~65% and 75% mortality was recorded with the application of 250 and 500 IJs per larva, respectively [100].

However, the persistence and efficacy of ENs are conditioned by abiotic factors such as ultraviolet light, extreme temperatures, and soil moisture content, pH, and texture, among others [101,102,103], and by biotic factors associated with the habitat. These include the availability and abundance of hosts and the interaction with their natural enemies in the form of pathogenic microorganisms (fungi) and microarthropods, such as hematophagous mites [103]. These cause a significant reduction in the abundance of IJs, raising questions regarding their efficacy. Adverse temperatures affect the oxygen exchange, persistence, survival, and dispersal of nematodes, and thus limit their infective capacity [104]. In addition, nematodes are also sensitive to low soil moisture content and extreme temperatures, and therefore, all these abiotic factors must be considered fundamental to ensure higher survival in the field for successful pest suppression [104,105].

A study under field conditions using concentrations of 5000, 1500, 500, and 150 IJs/cm^2^ showed that *S. feltiae* has a high infective capacity as it caused high mortality in the larvae of *C. capitata* [95]. However, other strains of this nematode species have caused high mortality at a lower IJ density. It has also been observed that a density of approximately 1000 IJs of *S. feltiae* per 3 dL of soil causes an average mortality of 58% under controlled conditions, while a density of 10,000 IJs per 3 dL of soil causes a mortality of 96%. This is a significantly higher mortality than that observed in the presence of entomopathogenic fungi and bacteria in soils without nematodes [74]. However, the foraging strategies of IJs, which are classified into ambushers and cruisers, may also influence the selection of nematodes for pest control programs.

Nematode concentration is closely associated with the foraging activity of species of the genus *Heterorhabditis* [105] since a mortality of more than 50% was reported in the larvae of five white grub species (Coleoptera: Scarabeidae) following the application of 30,000 IJs of *Heterorhabditis* sp. in soil [106]. It should be noted that this amount of soil is lower than that used in evaluations with fruit flies, but this example helps to emphasize the need to apply higher densities of IJs to achieve higher mortality in soil pests.

It has also been observed that many larvae removed from fruits exposed to IJs have not been infected, which suggests that the larvae were late third instars, and the nematodes were not able to infect them at this developmental stage [44]. This occurs because, once the puparium is formed, the hard exoskeleton acts as a protective cover, as spiracles, which are natural openings for infection, practically close and are surrounded by small spinnerets that act as a barrier that prevents penetration by IJs. Therefore, if the IJs encounter their host in the pupal stage, they cannot penetrate inside its body [79].

IJs have high ability to infect fruit fly larvae in sandy clay soil, with higher mortality when the moisture content is between 15 and 25%. It has been reported that inoculations of 1.2 to 2.5 billion nematodes/ha produce satisfactory results in terms of controlling larvae of coleopteran and dipteran pests [101].

To apply IJs for pest suppression purposes, the first step is to consider the availability of the infective agents and the cost associated with their production. However, if we consider that large-scale production has already been developed to produce large quantities of IJs at low cost, it only remains to define and optimize a viable route for their application in the environments where the pest is located, mainly where an area-wide integrated management program is carried out. Infective juveniles have been applied in this way to regulate populations of larvae or pupae of the citrus root weevil, *Diaprepes abbreviates*, and the plum curculio (*Conotrachelus nenuphar* (Herbst) [23,107].

Field studies on the use of IJs against fruit flies are limited and an adequate application strategy has yet to be defined. However, the results obtained regarding the use of IJs against the main fruit fly species [108], and the prevailing interest in generating more sustainable strategies suggest that there is an increasing use of IJs for pest control, and their application for the control of fruit flies should therefore be promoted in orchards, such as mango, citrus, guava, sapodilla, where these flies cause damage. Infective juveniles of ENs should be applied on the soil mainly in the drip area of “trap trees” within marginal areas, which exhibit high levels of damage caused by fruit flies, in line with the criteria used to control the plum weevil, *C. nenuphar* [107]. The knowledge generated provides the basic elements to integrate a strategy for the application of ENs in the field and to reduce the use of pesticides for pest control. However, the use of IJs will be enhanced with additional knowledge about the potential they offer to suppress fruit fly populations under field conditions in different environments and that will help to define the best form of application in the field. This approach could be used against fruit flies and contribute to the production of fruits and vegetables with less pesticide residues.

 **(b)**  **Biotic factors**

Among the main biotic factors that affect the efficacy of nematodes, fungi and bacteria are the most important. An antagonistic effect of these organisms has been observed on nematodes, as they cause lethal diseases and thus significantly reduce the number of effective IJs per soil area [109]. Nematophagous organisms such as mites and insects may limit the survival of entomopathogenic nematodes applied in the field [103]. The reduction in IJs by natural enemies and their limited dispersal capacity questions the effectiveness of these organisms as biological control agents, and thus it may sometimes be necessary to carry out additional applications for better pest control [110].

The constant presence of hosts is an important factor for the persistence of IJs in the soil. In this sense, tropical and subtropical environments can constantly provide insect hosts that guarantee a permanent population of IJs [111]. Therefore, considering that fruit fly larvae use the soil to pupate and that mango, citrus, guava, and sapodilla, among other orchards, are located in tropical and subtropical regions where temperatures are warm and humidity is high, combined with a predominance of soils with clayey loam and sandy clayey textures, a control program for these pests can be implemented by applying nematodes as an additional control agent, which would result in less environmental pollution caused by the conventional pesticides that are currently applied.

**Figure 2:**
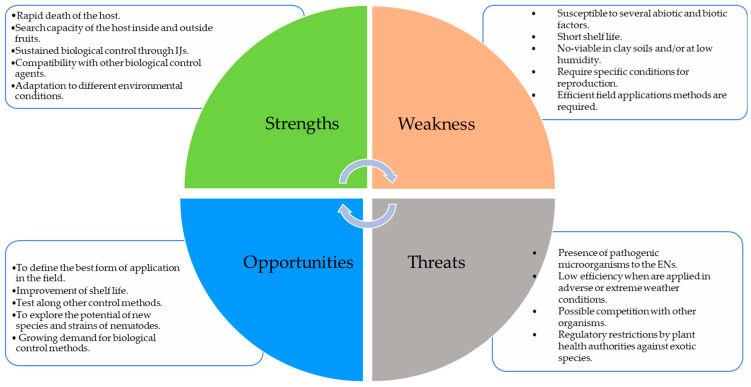
Factors that promote and limit the use of entomopathogenic nematodes for their application against fruit flies.

Considering the advantages and disadvantages that entomopathogenic nematodes face against fruit flies, areas of opportunity are identified to make their use more effective in a management program (Figure 2). An analysis was performed to determine the strengths, opportunities, weaknesses, and threats (SWOT) on the use of EPNs. Then, we can see that nematodes face several weaknesses, but there are also several opportunities that we should investigate to improve their performance, mainly under field conditions.

## 4. Future Perspective

To date, a total of 70 scientific articles have been published in 15 countries, which report different results that generally indicate a high level of ENs infection against fruit fly larvae and pupae demonstrating their potential as control agents against these pests. Additionally, a total of 32 species of ENs have been evaluated against 18 fruit fly species (Table 1). The study of this topic will continue in a dynamic way, focused mainly against the most economically important species such as *C. capitata* [112,113,114,115,116,117], *B. oleae* [118,119], *B. dorsalis* [120], *R. cerasi* and *A. fraterculus* [121]. The exploration of new species of nematodes continues and bioassays are carried out against the different biological stages of fruit flies [115]. It is also necessary to explore the most effective routes for its application in the field [107].

Currently, more than a dozen commercial EN strains have been developed and commercialized, globally, using 13 EN species. As mentioned above, ENs can be produced in vivo (by infecting susceptible insects), in vitro on solid media, and in vitro liquid media. The last two techniques require the culture of the symbiotic bacteria, before inoculating the IJs. The latter technique is the most efficient and cost/benefit accepted, in spite of the requirement of initial large investments. Still, a number of advancements can be made to improve production efficiency and biocontrol potential. For example, in vitro production can be improved by optimizing the culture media and by expanding the fermentation processing of the symbiotic bacteria. Additionally, the development of programs focused on finding improved nematode strains and the stabilization of beneficial traits, related to the obtain a stable production of ENs, is highly recommended. These are some of the main constraints in the productions of commercial EN products [122].

On the other hand, the short shelf-life of their commercial products may be the most important limitation to expand the commercial use of ENs. ENs must be prepared in an adequate formulation that should keep IJs under a humid and aeriated environment to keep them alive for an acceptable time. Many efforts have been carried out on this constrain and ideas such as the use of clay pellets with humid cores or, more recently, the use of gels balls (i.e., alginate capsules), have improved the shelf-life of these products. However, more research is required to make a real advancement, such as the development of methods focused to obtain dormant individuals which can be recovered before use. The specific use of ENs in fruit fly control has the interesting alternative of using fruits infested with fruit fly larvae, previously treated with IJs, and then, scattered under the dripping area of fruit trees [59].

**Table 1:** Species of entomopathogenic nematodes evaluated against several species of fruit flies in different experimental arenas.

Fruit Flies Species	Nematodes Entomopathogenic Species Tested	*N* ^1^	References
*Anastrepha fraterculus*	*Heterorhabditis bacteriophora*	1	Barbosa-Negrisoli et al., 2009 [97] Foekel et al., 2016 [37] Foekel et al., 2017 [38] Chaneiko et al., 2021 [65]
	*H. chongmingensis*	1	
	*H. amazonensis*	1	
	*Heterorhabditis* sp.	1	
	*Steinernema feltiae*	2	
	*S. carpocapsae*	2	
	*S. glaseri*	1	
	*S. riobrave*	1	
	*S. rarum*	1	
	*Steinernema* sp.	2	
	*Oscheius* sp.	2	
*A. ludens*	*H. bacteriophora*	1	Lezama-Gutiérrez et al., 1996 [99]. Lezama-Gutiérrez et al., 2006 [80]. Toledo et al., 2005 [32]. Toledo et al., 2006 [14]. Toledo et al., 2014 [76]
	*S. carpocapsae*	1	
	*S. feltiae*	1	
	*S. glaseri*	1	
	*S. riobrave*	1	
*A. serpentina*	*H. bacteriophora*	1	Toledo et al., 2006 [98]
*A. suspensa*	*H. bacteriophora*	1	Beavers & Calkin., 1984 [93]. Have et al., 2017 [9] Have et al., 2018 [10]
	*H. indica*	1	
	*H. floridensis*	1	
	*H. heliothidis*	1	
	*H. zealandica*	1	
	*S. carpocapsae*	1	
	*S. feltiae*	1	
	*S. glaseri*	2	
	*S. riobrave*	1	
	*S. rarum*	1	
	*S. diaprepesi*	1	
*A. obliqua*	*H. bacteriophora*	1	Toledo et al., 2005 [31] Toledo et al., 2009 [75]
	*S. carpocapsae*	1	
*Bactrocera dorsalis*	*H. indica*	1	Godjo et al., 2018 [13] Godjo et al., 2021 [120] Menzler-Hokkanen et al., 2022 [74]. Usman et al., 2021 [43] Wakil et al., 2022 [70]
	*H. taysearae*	1	
	*Heterorhabditis* sp.	1	
	*S. feltiae*	1	
	*S. taysearae*	1	
	*S. kandii*	2	
*B. latifrons*	*S. siamkayai*	1	Ta-Oun et al., 2022 [78].
*B. oleae*	*H. bacteriophora*	2	Sirjani et al., 2009 [123]. Torrini et al., 2017 [118]. Torrini et al., 2020 [119].
	*H. marelatus*	1	
	*S. carpocapsae*	2	
	*S. feltiae*	1	
	*S. glaseri*	1	
	*S. riobrave*	1	
*B. tryoni*	*H. bacteriophora*	1	Langford et al., 2014 [86].
	*S. carpocapsae*	1	Aryal et al., 2022 [124]
	*S. feltiae*	2	Attalla & Eweis, 2002 [63] Usman et al., 2021 [43].
*B. zonata*	*H. bacteriophora*	2	Mohmoud & Mohamed-Osman 2007 [125] Fetoh et al., 2011 [64] Nouh & Hussein 2014 [126]
	*S. carpocapsae*	2	
	*S. feltiae*	1	
*Ceratitis capitata*	*H. bacteriophora*	6	Almeida et al., 2007 [71]. Shaurub et al., 2015 [114]. Minas et al., 2016 [68]. Nouh & Hussein 2014 [126] Abdel-Razek & Abd-Elgawad 2021 [69] Kaprananas et al., 2021 [45] Yağcı1 et al., 2021 [116] Lindegren & Vail 1986 [94] Lindegren et al., 1990 [95] Kapranas et al., 2023 [117] Gazit et al., 2000 [67] Kepenekci & Susurluk 2006 [127] Karagoz et al., 2009 [8]. Malan & Manrakhan 2009 [40]. James et al., 2018 [81].
	*H. indica*	1	
	*H. zealandica*	1	
	*H. baujardi*	1	
	*H. noenieputensis*	1	
	*H. megidis*	1	
	*H. downesi*	1	
	*Heterorhabditis* sp.	2	
	*S.* (*Neoplactana*) *carpocapsae*	1	
	*S. carpocapsae*	7	
	*S. feltiae*	6	
	*S. riobrave*	1	
	*S. brazilense*	1	
	*S. weiseri*	1	
	*S. yirgalemense*	1	
	*S. khoisanae*	1	
	*Oscheius tipulae*	1	
*C. rosa*	*H. bacteriophora*	1	Malan & Manrakhan 2009 [40]
	*H. zealandica*	1	
	*S. khoisanae*	1	
*Dacus cialiatus*	*H. bacteriophora*	3	Fetoh & El-Gendi 2006 [36] Fetoh et al., 2011 [64] Kamali et al., 2013 [85]
	*S. carpocapsae*	3	
	*S. riobrave*	1	
*D. curcubitae*	*S. feltiae*	1	Lindegren & Vail 1986 [94]
*D. dorsalis*	*S. feltiae*	1	Lindegren & Vail 1986 [94]
*Rhagoletis cerasi*	*H. bacteriophora*	1	Kepenekci & Susurluk 2006 [127] Kepenekci et al., 2015 [121]
	*H. marelatus*	1	
	*S. carpocapsae*	1	
	*S. feltiae*	1	
*R. indifferens*	*H. bacteriophora*	1	Yee & Lacey 2003 [128] Patterson & Lacey 1999 [129]
	*H. marelatus*	1	
	*S. carpocapsae*	1	
	*S. feltiae*	1	
	*S. riobrave*	1	
	*S. intermedium*	1	
*R. pomonella*	*H. bacteriophora*	1	Poinar Jr et al., 1977 [53] Usman et al., 2020a [41] Usman et al., 2020b [42]
	*H. indica*	1	
	*S.* (*Neoaplectana*) sp.	1	
	*S. carpocapsae*	2	
	*S. feltiae*	1	
	*S. riobrave*	2	

*N* ^1^ = Frecuency of tested.

## 5. Conclusions

Based on the results reported to date, the larval phase appears to be the most susceptible biological stage to attacks by IJs, followed by the adult stage, while the pupal stage is the least susceptible one. The most frequent studies conducted to date have focused on determining whether IJs of different species have the potential to infect and kill fruit flies.

Considering the results obtained in different studies carried out under both laboratory and field conditions, it has been demonstrated that ENs have the potential to infect and kill larvae and adults of fruit flies to be considered effective biological control agents of these pests. Infective juveniles could be applied using a backpack sprayer, boom sprayer, microjet irrigation systems, trunk-sprayers, subsurface injection, or baits. However, it is necessary to know the physical and chemical properties of the soil where the fruit orchards are established for an optimal IJ performance. In this way, IJs can be more efficient at searching for and finding the different life stages of fruit flies that may exist in the tree drip area where they are less likely to be affected by conventional chemical insecticides. It has also been observed that the efficacy of these organisms is directly related to the texture, moisture content, and temperature of the substrate and, to a lesser extent, to host species and age. Interestingly, IJs applied directly on the soil can cause a high mortality of larvae when these leave the fruit to pupate in the soil and while they are still inside the fruit. Although the mortality caused by nematodes in larvae inside the fruit is lower, the application may be effective in domestic backyard trees, abandoned orchards, or fruit fly host trees located in marginal areas where no other control methods are used. Further studies are required to develop an effective method of application in the field that will provide the highest level of pest suppression.

## Data Availability

No new data were created, or all data cited are available. Therefore, authorization is not required.

## References

[1] Norrbom A.L., Kim K.C. (1988). A List of the Reported Host Plants of the Species of Anatrepha (Díptera: Tephritidae).

[2] Drew R.A.I., Hancock D.L. (1994). The *Bactrocera dorsalis* complex of fruit flies (Diptera: Tephritidae: Dacinae) in Asia. Bull. Entomol. Res. Suppl. Ser..

[3] Hernández-Ortiz V. (1992). El Género Anastrepha Schiner en México (Diptera: Tephritidae): Taxonomía, Distribución y sus Plantas Huéspedes.

[4] White I.M., Elson-Harris M.M. (1992). Fruit Flies of Economic Significance: Their Identification and Bionomics.

[5] Ekesi S., De Meyer M., Mohamed S.A., Virgilio M., Borgemeister C. (2016). Taxonomy, ecology, and management of native and exotic fruit fly species in Africa. Annu. Rev. Entomol..

[6] Reyes J., Santiago G., Hernández P., Tan K.H. (2000). The Mexican fruit fly programme. Area-Wide Control of Fruit Flies and Other Insect Pests.

[7] Enkerlin W., Ruelas J.G., Pantaleon R., Litera C.S., Cortés A.V., López J.Z., Dávila D.O., Gerardo P.M., Villarreal L.S., Roldán E.C. (2017). The Moscamed Regional Programme: Review of a success story of area-wide sterile insect technique application. Entomol. Exp. Appl..

[8] Karagoz M., Gulcu B., Hazir C., Kaya H.K., Hazir S. (2009). Biological control potential of Turkish entomopathogenic nematodes against the Mediterranean fruit fly *Ceratitis capitata*. Phytoparasitica.

[9] Heve W.K., El-Borai F.E., Carrillo D., Duncan L.W. (2017). Biological control potential of entomopathogenic nematodes for management of Caribbean fruit fly, *Anastrepha suspensa* Loew (Tephritidae). Pest Manag. Sci..

[10] Heve W.K., El-Borai F.E., Carrillo D., Duncan L.W. (2018). Increasing entomopathogenic nematode biodiversity reduces efficacy against the Caribbean fruit fly *Anastrepha suspensa*: Interaction with the parasitoid *Diachasmimorpha longicaudata*. J. Pest Sci..

[11] Heve W.K., El-Borai F.E., Johnson E.G., Carrillo D., Crow W.T., Duncan L.W. (2018). Responses of *Anastrepha suspensa*, *Diachasmimorpha longicaudata*, and sensitivity of guava production to *Heterorhabditis bacteriophora* in fruit fly integrated pest management. J. Nematol..

[12] Heve W.K., Adjadeh T.A., Billahc M.K. (2021). Overview and future research needs for development of effective biocontrol strategies for management of *Bactrocera dorsalis* Hendel (Diptera: Tephritidae) in sub-Saharan Africa. Pest Manag. Sci..

[13] Godjo A., Zadji L., Decraemer W., Willems A., Afouda L. (2018). Pathogenicity of indigenous entomopathogenic nematodes from Benin against mango fruit fly (*Bactrocera dorsalis*) under laboratory conditions. Biol. Control.

[14] Toledo J., Rasgado M.A., Ibarra J.E., Gómez A., Liedo P., Williams T. (2006). Infection of *Anastrepha ludens* following soil aplications of *Heterorhabditis bacteriophora* in a mango orchard. Entomol. Exp. Appl..

[15] Toledo J., Flores S., Campos S., Villaseñor A., Enkerlin W., Liedo P., Valle Á., Montoya P. (2017). Pathogenicity of three formulations of *Beauveria bassiana* and efficacy of autoinoculation devices and sterile fruit fly males for dissemination of conidia for the control of *Ceratitis capitata*. Entomol. Exp. Appl..

[16] Montoya P., Toledo J., Montoya P., Toledo J., Hernández E. (2020). Estrategias de control biológico. Moscas de la Fruta: Fundamentos y Procedimientos Para su Manejo.

[17] Dolinski C., Lacey L.A. (2007). Microbial control of arthropod pests of tropical tree fruits. Neotrop. Entomol..

[18] Dolinski C. (2016). Entomopathogenic nematodes against the main guava insect pests. BioControl.

[19] Kaya H.K., Hoy M.A., Herzog D.C. (1985). Entomogenous nematodes for insect control in IPM systems. Biological Control in Agricultural IPM Systems.

[20] Triggiani O., Poinar G.O. (1976). Infection of adult Lepidoptera by *Neoaplectana carpocapsae* (Nematoda). J. Invertebr. Pathol..

[21] Woodring J.L., Kaya H.K. (1988). Steinernematid and Heterorhabditid Nematodes: A Handbook of Biology and Techniques.

[22] Lewis E.E., Gaugler R. (2002). Behavioral ecology. Entomopathogenic Nematology.

[23] Stuart R.J., El-Borai F.E., Duncan L.W. (2008). From augmentation to conservation of entomopathogenic nematodes: Trophic cascades, habitat manipulation and enhanced biological control of *Diaprepes abbreviatus* root weevils in Florida citrus groves. J. Nematol..

[24] Jansson R.K., Lecrone S.H., Gaugler R. (1993). Field efficacy and persistence of entomopathogenic nematodes (Rhabditida: Steinernematidae, Heterorhabditidae) for control of sweetpotato weevil (Coleoptera: Apionidae) in southern Florida. J. Econ. Entomol..

[25] Klein M.G., Bedding R., Akhurst R., Kaya H. (1993). Biological control of scarabs with entomopathogenic nematodes. Nematodes and Biological Control of Insect Pest.

[26] Glazer Y., Navon A. (1990). Activity and persistence of entomoparasitic nematodes tested againt *Heliothis armigera* (Lepidoptera: Noctuidae). J. Econ. Entomol..

[27] Adams B.J., Fodor A., Koppenhofer H.S., Stackebrandt E., Stock S.P., Klein M.G. (2006). Biodiversity and systematics of nematode-bacterium entomopathogens. Biol. Control.

[28] Mullens B.A., Meyer J.A., Cyr T.L. (1987). Infectivity of insect-parasitic nematodes (Rhabditida: Steinernematidae, Heterorhabditidae) for larvae of some Manure-breeding flies (Diptera: Muscidae). Environ. Entomol..

[29] Chen S., Han X., Moens M. (2003). Effect of temperature on the pathogenicity of entomopathogenic nematodes (Steinernema and Heterorhabditis spp.) to *Delia radicum*. BioControl.

[30] Navarro M.J., Gea F.J. (2014). Entomopathogenic nematodes for the control of phorid and sciarid flies in mushroom crops. Pesqui. Agropecuária Bras..

[31] Toledo J., Martinez C., Liedo P., Ibarra J.E. (2005). Susceptibilidad de larvas de *Anastrepha obliqua* Macquart (Diptera: Tephritidae) a Heterorhabditis bacteriophora (Poinar) (Rhabditidae: Heterorhabditidae) en condiciones de laboratorio. Vedalia.

[32] Toledo J., Ibarra J.E., Liedo P., Gomez A., Rasgado M.A., Williams T. (2005). Infection of *Anastrepha ludens* (Diptera: Teprhitidae) larvae by *Heterorhabditis bacteriophora* (Rhabditida: Heterorhabditidae) under laboratory and field conditions. Biocontrol Sci. Technol..

[33] Diksha, Mahajan E., Singh S., Kaur-Sohal S. (2022). Potential biological control agents of *Zeugodacus cucurbitae* (Coquillett): A review. J. Appl. Entomol..

[34] Shaurub E.H. (2023). Review of entomopathogenic fungi and nematodes as biological control agents of tephritid fruit flies: Current status and a future vision. Entomol. Exp. Appl..

[35] Orozco-Dávila D., Quintero L., Hernández E., Solís E., Artiaga T., Hernández R., Ortega C., Montoya P. (2017). Mass rearing and sterile insect releases for the control of *Anastrepha* spp. pests in Mexico—A review. Èntomol. Exp. Appl..

[36] Fetoh B.E.A., El-Gendi S.S. (2006). Impact of entomopathogenic nematodes on different stages of the pumpkin fly, *Dacus ciliatus* as a new approach in its biological control. Fayoum J. Agric. Res. Dev..

[37] Foelkel E., Monteiro L.B., Voss M. (2016). Virulence of nematodes against larvae of the south American fruit fly in laboratory using soil from Porto Amazonas, Paraná, Brazil, as substrate. Ciência Rural.

[38] Foelkel E., Voss M., Monteiro L.B., Nishimura G. (2017). Isolation of entomopathogenic nematode in an apple orchard in southern Brazil and its virulence to *Anastrepha fraterculus* (Diptera: Tephritidae) larvae, under laboratory conditions. Braz. J. Biol..

[39] Abbas M.S.T., Nouh-Gehan M., Abdel-Samad S.S.M., Negm A.A. (2016). Infectivity of the entomopathogenic nematodes as bio-control agents to *Spodoptera littorals*, *Ceratitis capitata* and *Bactrocera zonata*. Egypt. J. Biol. Pest Control.

[40] Malan A.P., Manrakhan A. (2009). Susceptibility of the Mediterranean fruit fly (*Ceratitis capitata*) and the Natal fruit fly (*Ceratitis rosa*) to entomopathogenic nematodes. J. Invertebr. Pathol..

[41] Usman M., Gulzar S., Wakil W., Wu S., Piñero J.C., Leskey T.C., Nixon L.J., Oliveira-Hofman C., Toews M.D., Shapiro-Ilan D. (2020). Virulence of entomopathogenic fungi to *Rhagoletis pomonella* (Diptera: Tephritidae) and interactions with entomopathogenic nematodes. J. Econ. Entomol..

[42] Usman M., Gulzar S., Wakil W., Piñeiro J.C., Leskey T.C., Nixon L.J., Oliveira-Hofman C., Wu S., Shapiro-Ilan D. (2020). Potential of entomopathogenic nematodes against the pupal stage of the apple maggot *Rhagoletis pomonella* (Walsh) (Diptera: Tephritidae). J. Nematol..

[43] Usman M., Wakil W., Shapiro-Ilan D.I. (2021). Entomopathogenic nematodes as biological control agent against *Bactrocera zonata* and *Bactrocera dorsalis* (Diptera: Tephritidae). Biol. Control.

[44] Herrera–Aguilar J. (2013). Mortalidad de Larvas de *Anastrepha obliqua* (Diptera. Tephritidae) con el Nematodo *Heterorhabditis bascteriophora* en Suelo Areno-Arcilloso. Bachelor’s Thesis.

[45] Kapranas A., Chronopoulou A., Lytra I.C., Peters A., Milonasa P.G., Papachristos D.P. (2021). Efficacy and residual activity of commercially available entomopathogenic nematode strains for Mediterranean fruit fly control and their ability to infect infested fruits. Pest Manag. Sci..

[46] Herz A., Köppler K., Vogt H., Elias E., Katz P., Peters A. (2006). Biological control of the cherry fruit fly, *Rhagoletis cerasi* L. (Diptera: Tephritidae) by use of entomopathogenic nematodes: First experiences towards practical implementation. Proceedings of the Conference Ecofruit—12th International Conference on Cultivation Technique and Phytopathological Problems in Organic Fruit-Growing.

[47] Shapiro-Ilan D.I., Gaugler R., Tedders W.L., Brown I., Lewis E.E. (2002). Optimization of inoculation for in vivo production of entomopathogenic nematodes. J. Nematol..

[48] Wouts W.M. (1981). Mass production of the entomogenous nematode, *Heterorhabditis heliothidis* (Nematoda: Heterorhabditidae) on artificial media. J. Nematol..

[49] Bedding R.A. (1981). Low cost in vitro mass production of *Neoaplectana* and *Heterorhabditis* species (Nematoda) for field control of insect pests. Nematologica.

[50] Ehlers R.U. (2001). Mass production of entomopathogenic nematodes of plant protection. Appl. Microbiol. Biotechnol..

[51] Lindegren J.E., Valero K.A., Mackey B.E. (1993). Simple in vivo production and storage methods for *Steinernema carpocapsae* infective juveniles. J. Nematol..

[52] Peters A., Han R., Yan X., Leite L.G. (2017). Production of entomopathogenic nematodes. Microbial Control of Insect and Mite Pests.

[53] Poinar G.O., Thomas G.M. (1966). Significance of *Achromobacter nematophilus* sp. nov. (Achromobacteriaceae: Eubacteriales) asso-ciated with a nematode. Int. Bull. Bacteriol. Nomencl. Taxon..

[54] Maciel-Vergara G., Rodríguez-Hernández A.I., Chavarría-Hernández N. (2010). Cultivo monoxénico sumergido del nematodo entomopatógeno, *Steinernema carpocapsae* CABA01, en biorreactor airlift con recirculación interna. Bio Tecnol..

[55] Cho C.H., Whang K.S., Gaugler R., Yoo S.K. (2011). Submerged monoxenic culture medium development for *Heterorhabditis bacteriophora* and its symbiotic bacterium *Photorhabdus luminescens*: Protein sources. J. Microbiol. Biotechnol..

[56] Dunn M.D., Belur P.D., Malan A.P. (2021). A review of the in vitro liquid mass culture of entomopathogenic nematodes. Biocontrol Sci. Technol..

[57] Devi G. (2018). Mass production of entomopathogenic nematodes—A review. Int. J. Environ. Agric. Biotechnol..

[58] Shapiro-Ilan D.I., Gaugler R. (2002). Production technology for entomopathogenic nematodes and their bacterial symbionts. J. Ind. Microbiol. Biotechnol..

[59] Shapiro-Ilan D.I., Han R., Qiu X., Morales-Ramos J.A., Rojas M.G., Shapiro-Ilan D.I. (2014). Production of entomopathogenic nematodes. Mass Production of Beneficial Organisms.

[60] Gaugler R., Brown I. (2001). Apparatus and Method for Mass Production of Insecticidal Nematodes. U.S. Patent.

[61] Young J.M., Dunnill P., Pearce J.D. (2002). Separation characteristics of liquid nematode cultures and the design of recovery operations. Biotechnol. Prog..

[62] Tabassum K.A., Shahina F. (2004). In vitro mass rearing of different species of entomopathogenic nematodes in monoxenic solid culture. Pak. J. Nematol..

[63] Attalla F.A., Eweis M.A. (2002). Preliminary investigation on the utilization of entomopathogenic nematodes as biological control agents against the peach fruit fly, *Bactrocera zonata* (Saunders) (Diptera: Tephritidae). Egypt. J. Agric. Res..

[64] Fetoh B.E.S.A., Abdel-Gawad A.A., Shalaby F.F., Elyme M.F. (2011). Pathogenic and lethal effects of the entomopathogenic nematodes on the peach fruit fly, *Bactrocera zonata* (Saunders) and the cucurbit fruit fly, *Dacus ciliatus* (Loew) (Diptera: Tephritidae). Egypt. J. Agric. Res..

[65] Chaneiko S.M., de Brida A.L., Bernardi D., Leite L.G., Garcia F.R.M. (2021). Biological activity of entomopathogenic nematodes on *Anastrepha fraterculus* (Diptera: Tephritidae). Biosci. J..

[66] Jean-Baptiste M.C., Lima de Brida A., Bernardi D., da Costa Días S., Pazini J.B., Leite L.G., Siciliano-Wilcken S.R., Mello-Garcia F.R. (2021). Effectiveness of entomopathogenic nematodes against *Ceratitis capitata* (Diptera: Tephritidae) pupae and nematode compatibility with chemical insecticides. J. Econ. Entomol..

[67] Gazit Y., Rosler Y., Glazer I. (2000). Evaluation of entomopathogenic nematodes for the control of Mediterranean fruit fly (Diptera: Tephritidae). Biocontrol Sci. Technol..

[68] Minas R.S., Moreira-Souza R., Dolinski C., da Silva-Carvalho R., da Silva-Burla R. (2017). Potential of entomopathogenic nematodes (Rhabditida: Heterorhabditidae) to control Mediterranean fruit fly (Diptera: Tephritidae) soil stages. Nematoda.

[69] Abdel-Razek A.S., Abd-Elgawad M.M.M. (2021). Spinosad combined with entomopathogenic nematode for biocontrol of the Mediterranean fruit fly (*Ceratitis capitata* [Wiedemann]) on citrus. Egypt J. Biol. Pest Control.

[70] Wakil W., Usman M., Piñero J.C., Wu S., Toews M.D., Shapiro-Ilane D.I. (2022). Combined application of entomopathogenic nematodes and fungi against fruit flies, *Bactrocera zonata* and *B. dorsalis* (Diptera: Tephritidae): Laboratory cups to field study. Pest Manag. Sci..

[71] Almeida J.A.M., Batista-Filho A., Oliveira F.C., Raga A. (2009). Pathogenicity of the entomopathogenic fungi and nematode on medfly *Ceratitis capitata* (Wied.) (Diptera: Tephritidae). BioAssay.

[72] Gava C.A.T., Paranhos B.A.J. (2023). Combining the virulent *Beauveria bassiana* (Balsam) Vuillemin LCB289 and nematode strains to control pupae of *Ceratitis capitata* Wiedemann. Biocontrol Sci. Technol..

[73] Medina P., Corrales E., González-Nuñez M., Smagghe G., Viñuela E. (2008). Effects of *Beauveria bassiana*, *Heterorhabditis bacteriophora*, *H. megidis* and *Steinernema feltiae* on the Mediterranean fruit fly *Ceratitis capitata* and the very sensitive braconid *Psyttalia concolor* in the lab. IOBC/Wprs Bull..

[74] Menzler-Hokkanen I., Ruhanen H., Hokkanen H.M.T. (2022). Mortality of the oriental fruit fly, *Bactrocera dorsalis*, during pupation in insect pest suppressive soils. Entomol. Exp. Appl..

[75] Toledo J., Williams T., Pérez C., Liedo P., Valle J.F., Ibarra J.E. (2009). Abiotic factors affecting the infectivity *of Steinernema carpocapsae* (Rhabditida: Steinernematidae) on larvae of *Anastrepha obliqua* (Macquart) (Diptera: Tephritidae). Biocontrol Sci. Technol..

[76] Toledo J., Sánchez J.E., Williams T., Gómez A., Montoya P., Ibarra J.E. (2014). Effect of soil moisture in relation to *Heterorhabditis bacteriophora* persistence and efficacy against *Anastrepha ludens* larvae. Fla. Entomol..

[77] Rohde C., Moino A., Da Silva M.A.T., Carvalho F.D., Ferreira C.S. (2010). Influence of soil temperature and moisture on the infectivity of entomopathogenic nematodes (Rhabditida: Heterorhabditidae, Steinernematidae) against larvae of *Ceratitis capitata* (Wiedemann) (Diptera: Tephritidae). Neotrop. Entomol..

[78] Ta-un P., Ehlers R.-U., Nimkingrat P. (2022). Effects of soil texture and moisture on the host searching abilities of *Steinernema siamkayai* against *Bactrocera latifrons*. Nematology.

[79] Toledo J., Gurgúa J.L., Liedo P., Ibarra J.E., Oropeza A. (2001). Parasitismo de larvas y pupas de la mosca mexicana de la fruta, *Anastrepha ludens* (Loew) (Diptera: Tephritidae) por el nematodo *Steinernema feltiae* (Filipjev) (Rhabditidae: Steinernematidae). Vedalia.

[80] Lezama-Gutiérrez R., Molina-Ochoa J., Pescador-Rubio A., Galindo-Velasco E., Ángel-Sahagún C.A., Michel-Aceves A.C., González-Reyes E. (2006). Efficacy of Steinernematid nematodes (Rhabditida: Steinernematidae) on the suppression of *Anastrepha ludens* (Diptera: Tephritidae) larvae in soil of differing textures: Laboratory and field trials. J. Agric. Urban Entomol..

[81] James M., Malan A.P., Pia Addison P. (2018). Surveying and screening South African entomopathogenic nematodes for the control of the Mediterranean fruit fly, *Ceratitis capitata* (Wiedemann). Crop Prot..

[82] Jackson C.G., Long J.P., Klungness L.M. (1998). Depth of pupation in four species of fruit flies (Diptera: Tephritidae) in sand with and without moisture. J. Econ. Entomol..

[83] Hodgson P.J., Sivinski J., Quintero G., Aluja M. (1998). Depth of pupation and survival of fruit fly (*Anastrepha* sppTephritidae) Pupae in a Range of Agricultural Habitats. Environ. Entomol..

[84] Montoya P., Flores S., Toledo J. (2008). Effect of rainfall and soil moisture on survival of adults and immature stages of *Anastrepha ludens* and *A. obliqua* (Diptera: Tephritidae) under Semi-field Conditions. Fla. Entomol..

[85] Kamali S., Karimi J., Hosseini M., Campos-Herrera R., Duncan L.W. (2013). Biocontrol potential of the entomopathogenic nematodes *Heterorhabditis bacteriophora* and *Steinernema carpocapsae* on cucurbit fly, *Dacus ciliatus* (Diptera: Tephritidae). Biocontrol Sci. Technol..

[86] Langford E.A., Nielsen U.N., Johnson S.N., Riegler M. (2014). Susceptibility of Queensland fruit fly, *Bactrocera tryoni* (Froggatt) (Diptera: Tephritidae), to entomopathogenic nematodes. Biol. Control.

[87] Poinar G.O., Thomas G., Prokopy R.J. (1977). Microorganisms associated with *Rhagoletis pomonella* (Diptera: Tephritidae) in Massachusetts. Proc. Entomol. Soc. Ont..

[88] Toledo A.J. (2002). Evaluación de Algunos Agentes Entomopatogenos Para el Control Microbiano de tres Especies de Moscas de la Fruta (Diptera: Teprhitidae) de Importancia Económica. Ph.D. Thesis.

[89] Alyokhin A.V., Mille C., Messing R.H., Duan J.J. (2001). Selection of pupation habitats by oriental fruit fly larvae in the laboratory. J. Insect Behav..

[90] Portillo-Aguilar C., Villani M.G., Tauber M.J., Tauber C.A., Nyrop J.P. (1999). Entomopathogenic nematode (Rhabditida: Heterorhabditidae and Steinernematidae) response to soil texture and bulk density. Environ. Entomol..

[91] Georgis R., Poinar G.O. (1983). Effect of soil texture on the distribution and infectivity of *Neoaplectana carpocapsae* (Nematoda: Steinernematidae). J. Nematol..

[92] Kaya H.K., Samson R.A., Vlak J.M., Peters D. (1986). Constraints associated with commercialization of entomogenous nematodes. Fundamental and Applied Aspects of Invertebrate Pathology.

[93] Beavers J.B., Calkin C.O. (1984). Susceptibility of *Anastrepha suspensa* (Diptera: Tephritidae) to Steinernematid and Heterorhabditid namatodes in laboratory studies. Environ. Entomol..

[94] Lindegren J.E., Vail P.V. (1986). Susceptibility of Mediterranean fruit fly, melon fly and oriental fruit fly (Diptera: Tephritidae) to the entomogenous nematode *Steinernema feltiae* in laboratory tests. Environ. Entomol..

[95] Lindegren J.E., Wong T.T., McInnis D.O. (1990). Response of Mediterranean fruit fly (Diptera: Tephritidae) to the entomogenous nematode *Steinernema feltiae* in field tests in Hawaii. Environ. Entomol..

[96] Gurgua J.L., Liedo P., Ibarra J.E., Oropeza A., Toledo J. Efecto del suelo y la temperatura en el parasitismo del nematodo entomopatógenos, *Steinernema feltiae* (Filipjev) (Rhabditidae: Steinernematidae) en la mosca mexicana de la fruta. Proceedings of the XXII National Congress of Biological Control.

[97] Barbosa-Negrisoli C.R.C., Garcia M.S., Dolinski C., Negrisoli A.S., Bernardi D., Nava D.E. (2009). Efficacy of indigenous entomopathogenic nematodes (Rhabditida: Heterorhabditidae, Steinernematidae), from Rio Grande do Sul Brazil, against *Anastrepha fraterculus* (Wied.) (Diptera: Tephritidae) in peach orchards. J. Invertebr. Pathol..

[98] Toledo J., Rojas R., Ibarra J.E. (2006). Efficiency of *Heterorhabditis bacteriophora* (Nematoda: Heterorhabditidae) on *Anastrepha serpentina* (Diptera: Tephritidae) larvae under laboratory conditions. Fla. Entomol..

[99] Lezama G.R., Molina O.J., González R.M., Trujillo de la Luz A., Rebolledo O.D. (1996). Susceptibilidad de larvas de *Anastrepha ludens* (Diptera: Tephritidae) a diversos nematodos entomopatógenos (Steinernematidae y Heterorhabditidae). Vedalia.

[100] Loulou A., M’saad-Guerfali M., Muller A., Bhat A.H., Abolafia J., Machado R.A.R., Kallel S. (2022). Potential of *Oscheius tipulae* nematodes as biological control agents against *Ceratitis capitata*. PLoS ONE.

[101] Gaugler R. (1988). Ecological consideration in the biological control of soil-habitating insect with entomopathogenic nematode. Agric. Ecosyst. Environ..

[102] Geden C.J., Axtell R.C. (1988). Effect of temperature on nematode *Steinernema feltiae* (Nematoda: Steinernematidae) treatment of soil for control of laser mealworm (Coleoptera: Tenebrionidae) in turkey house. J. Econ. Entomol..

[103] Epsky N.D., Walter D.E., Capinera J.L. (1988). Potential role of nematophagous microartropods as biotic mortality factors of entomogenous nematodes (Rhabditida: Steinernematidae, Heterorhabditidae). J. Econ. Entomol..

[104] Kung S.P., Gaugler R. (1991). Effects of soil temperature, moisture, and relative humidity on entomopathogenic nematode persistence. J. Invertebr. Pathol..

[105] Georgis R., Gaugler R. (1991). Predictability in biological control using enthomophathogenic nematodes. J. Econ. Entomol..

[106] Koppenhöfer A.M., Grewal P.S., Fuzy E.M. (2006). Virulence of the entomopathogenic nematodes *Heterorhabditis bacteriophora*, *Heterorhabditis zealandica*, and *Steinernema scarabaei* against five white grub species (Coleoptera: Scarabaeidae) of economic importance in turfgrass in North America. Biol. Control.

[107] Shapiro-Ilan D.I., Wright S.E., Leskey T.C., Tuttle A.F., Cooley D.R., Leskey T.C. (2013). Using entomopathogenic nematodes for biological control of plum curculio, *Conotrachelus nenuphar*: Effects of irrigation and species in apple orchards. Biol. Control.

[108] Belien T. (2018). Entomopathogenic nematodes as biocontrol agents of insect pests in orchards. CABI Rev..

[109] Poinar G.O., Jansson H.B. (1986). Infection of *Neoaplectana* and *Heterhabditis* (Rhabditiae; Nematoda) with the predatory fungi, *Monacrospporium ellipsoporum* and *Artrobotrys oliogospora* (Monilliales: Deuteromyces). Rev. De Nematol..

[110] Wright R.J., Agudelo-Silva F., Georgis R. (1987). Soil applications of steinernematid and heterorhabditid nematodes for control of Colorado potato beetles, *Leptinotarsa decemlineata* (Say). J. Nematol..

[111] Capinera J.L., Epsky N.D. (1992). Potential for biological control of soils insects in the Caribbean basin using entomopathogenic nematode. Fla. Entomol..

[112] Da Silva A.C., Batista–Filho A., Leite L.G., Tavares F.M., Raga A., Schmidt F.S. (2010). Efeito de nematoides entomopatogênicos na mortalidade da mosca-do-Mediterrâneo, *Ceratitis capitata*, e do gorgulho-da-goiaba, *Conotrachelus psidii*. Nematol. Bras..

[113] Rohde C., Mertz N.R., Junior A.M. (2020). Entomopathogenic nematodes on control of Mediterranean fruit fly (Diptera: Tephritidae). Rev. Caatinga.

[114] Shaurub E.H., Soliman N.A., Hashem A.G., Abdel-Rahman A.M. (2015). Infectivity of four entomopathogenic nematodes in relation to environmental factors and their effects on the biochemistry of the Medfly *Ceratitis capitata* (Wied.) (Diptera: Tephritidae). Neotrop. Entomol..

[115] Mokrini F., Laasli S.E., Benseddik Y., Joutei A.B., Blenzar A., Lakhal H., Sbaghi M., Imren M., Özer G., Paulitz T. (2020). Potential of Moroccan entomopathogenic nematodes for the control of the Mediterranean fruit fly, *Ceratitis capitata* Wiedemann (Diptera: Tephritidae). Sci. Rep..

[116] Yağcı1 M., Tuğba Akdeniz Fırat T.A., Dolunay-Erdoğuş F., Şahin M. (2021). Virulence of four entomopathogenic nematode against different stages of the Mediterranean fruit fly, *Ceratitis capitata* Wiedemann (Diptera: Tephritidae). Egypt. J. Biol. Pest Control.

[117] Kapranas A., Anna Chronopoulou A., Peters A., Antonatos S., Lytra I., Milonas P., Papachristos D. (2023). Early and off-season biological control of medfly with entomopathogenic nematodes: From laboratory experiments to successful field trials. Biol. Control.

[118] Torrini G., Mazza G., Benvenuti C., Roversi P.F. (2017). Susceptibility of olive fruit fly, *Bactrocera oleae* (Diptera: Tephritidae) pupae to entomopathogenic nematodes. J. Plant Prot. Res..

[119] Torrini G., Mazza G., Benvenuti C., Simoncini S., Landi S., Frosinini R., Rocchini A., Roversi P. (2020). FEntomopathogenic nematodes as potential biocontrol agents against *Bactrocera oleae* (Diptera: Tephritidae). Biocontrol Sci. Technol..

[120] Godjo A., Chabi N., Zadji L., Dossou P., Batcho O., Baimey H., Bonou W., Sinzogan A.A.C., Bokonon-Ganta A., Decraemer W. (2021). Evaluation of the ability of indigenous nematode isolates of *Heterorhabditis taysearae* and *Steinernema kandii* to control mango fruit fly *Bactrocera dorsalis* under laboratory, semi-field and field conditions in Northern Benin. Crop Prot..

[121] Kepenekci I., Hazir S., Özdem A. (2015). Evaluation of native entomopathogenic nematodes for the control of the European cherry fruit fly *Rhagoletis cerasi* L. (Diptera: Tephritidae) Larvae Soil. Turk. J. Agric. For..

[122] Koppenhöfer A.M., Shapiro-Ilan D.I., Hiltpold I. (2020). Entomopathogenic nematodes in sustainable food production. Front. Sustain. Food Syst..

[123] Sirjani F.O., Lewis E.E., Kaya H.K. (2009). Evaluation of entomopathogenic nematodes against the olive fruit fly, *Bactrocera oleae* (Diptera: Tephritidae). Biol. Control.

[124] Aryal S., Nielsen U.N., Sumaya N.H., Wilson C., Riegler M. (2022). Virulence, penetration rate and reproductive potential of entomopathogenic nematodes from eastern Australia in Queensland fruit fly, *Bactrocera tryoni*. Biol. Control.

[125] Mahmoud M.F., El-Naeim-Mohamed-Osman M.A. (2007). Use of the nematode *Steinernema feltiae* Cross N 33 as a biological control agent against the peach fruit fly, *Bactrocera zonata*. Tunis. J. Plant Prot..

[126] Nouh G.M., Hussein M.A. (2014). The role of entomopathogenic nematodes as biocontrol agents against some Tephritid flies. Adv. Biol. Resear..

[127] Kepenekci I., Susurluk A. (2006). Infectivity of two Turkish isolates of *Steinernema feltiae* (Rhabditida: Steinernematidae) against *Rhagoletis cerasi* and *Ceratitis capitata*. Nematol. Mediterranea.

[128] Yee W.L., Lacey L.A. (2003). Stage-specific mortality of *Rhagoletis indifferens* (Diptera: Tephritidae) exposed to three species of *Steinernema* nematodes. Biol. Control.

[129] Patterson-Stark J.E., Lacey L.A. (1999). Susceptibility of western cherry fruit fly (Diptera: Tephritidae) to five species of entomopathogenic nematodes in laboratory studies. J. Invertebr. Pathol..

